# Stressing State Analysis of Reinforced Concrete Beam Strengthened by CFRP Sheet with Anchoring Device

**DOI:** 10.3390/ma14030576

**Published:** 2021-01-26

**Authors:** Liang Luo, Jie Lai, Jun Shi, Guorui Sun, Jie Huang, Maoguo Yuan

**Affiliations:** 1 School of Civil Engineering, Central South University, Changsha 410075, China; 201821008531@mail.scut.edu.cn; 2 School of Civil Engineering and Transportation, South China University of Technology, Guangzhou 510641, China; 3 Academy of Combat Support, Rocket Force University of Engineering, Xi’an 710025, China; dadalai1234@sina.com; 4 National Engineering Laboratory for High Speed Railway Construction, Changsha 410000, China; 5 Key Lab of Structures Dynamic Behavior and Control of the Ministry of Education, School of Civil Engineering, Harbin Institute of Technology, Harbin 150090, China; 19B933012@stu.hit.edu.cn (G.S.); 19S033047@stu.hit.edu.cn (J.H.); 6 Engineering Construction Limited Liability Company of Yi’nan County, Linyi 276300, China; yuanmaoguo_75@163.com

**Keywords:** stressing state, failure load, middle anchorage, end anchorage, reinforcement concrete beam

## Abstract

This paper investigates the working performance of reinforcement concrete (RC) beams strengthened by Carbon-Fiber-Reinforced Plastic (CFRP) with different anchoring under bending moment, based on the structural stressing state theory. The measured strain values of concrete and Carbon-Fiber-Reinforced Plastic (CFRP) sheet are modeled as generalized strain energy density (GSED), to characterize the RC beams’ stressing state. Then the Mann–Kendall (M–K) criterion is applied to distinguish the characteristic loads of structural stressing state from the curve, updating the definition of structural failure load. In addition, for tested specimens with middle anchorage and end anchorage, the torsion applied on the anchoring device and the deformation width of anchoring device are respectively set parameters to analyze their effects on the reinforcement performance of CFRP sheet through comparing the strain distribution pattern of CFRP. Finally, in order to further explore the strain distribution of the cross-section and analyze the stressing-state characteristics of the RC beam, the numerical shape function (NSF) method is proposed to reasonably expand the limited strain data. The research results provide a new angle of view to conduct structural analysis and a reference to the improvement of reinforcement effect of CFRP.

## 1. Introduction

Over the past several decades, Carbon-Fiber-Reinforced Plastic (CFRP) sheets or laminates that are lightweight, have high tensile strength, have non-corrosive characteristics, and assemble conveniently have been widely adopted for structural strengthening and repair to extend their service life [1,2,3]. Although the use of CFRP is now generally recognized as a practically efficient method to improve the load-carrying capacity and durability of damaged or deteriorating structures, it may still result in a lower ductility and possibly a brittle failure mode, due to the debonding of CFRP or sudden ripping of concrete [4,5,6]. Correspondingly, researchers have conducted extensive experimental researches and numerical simulations on the bond performance of CFRP to concrete interface [7,8,9], since the bond plays a significant role in transferring the stress between concrete and CFRP, to develop composite action [10,11]. The CFRP laminates or sheets were generally applied on the surfaces of the specimens to be strengthened. The relevant findings indicated that the expected reinforcement effect was hard to achieve and the full tensile strength of CFRP could not be utilized, due to premature peeling failure of CFRP [12,13,14], which also restricted the application of CFRP to some extent. 

In order to enhance the utilization efficiency of CFRP, postpone the structural debonding failure, and improve the capability and safety of reinforcement method, some approaches are proposed to resolve the peeling of CFRP from the concrete surface or delamination between CFRP layers. Ceroni et al. [15] studied the bond behavior, strength, and failure modes of concrete elements strengthened by eight types of end fixings. The experimental results confirmed the effectiveness of different schemes for end fixing of CFRP sheets. Grooving on the concrete surface could also be an alternative method of surface preparation to effectively develop the bond performance between CFRP and concrete, resulting in the improvement of ultimate load capacity of strengthened beams, compared to those lacking any surface preparation [16,17]. Hayder et al. [18] adopted distributed external U-wrap CFRP anchorage to strengthen the three identical T-beams and three identical rectangular beams. The results indicated that higher flexural capacity of tested beams could be achieved through using external U-wraps anchorage. Ferreira et al. [19] evaluated the influence of anchorage on the flexural strength of beams strengthened with CFRP sheets or laminates based on extensive experimental results and discussed the design criteria of ACI 440-2R. The findings verified that the results derived from theoretical models were conservative. This research has highly contributed to the application of CFRP in the field of structural repair and strengthening. Meanwhile, Benvenuti and other researchers [20,21], based on the regularization mechanism, proposed the extended finite element model (XFEM), focusing on the separation process and the crack model, revealing the advantages of the middle bending crack in the peeling, and explaining the concrete and the interaction between FRP sheets and steel bars.

However, most researchers’ research on CFRP-strengthened beams seems to focus on the increase of the ultimate load capacity, rather than elucidating the changes in the working performance of the specimen during the entire load process that cannot be effectively revealed [22,23]. In addition, previous works in the literature have proposed many methods to prevent the debonding of CFRP sheets, but the research on improving the utilization efficiency of CFRP anchoring systems is far from enough. The working performance and utilization effect of the anchoring system have not yet been essentially quantified, which also requires a lot of experimental research and theoretical analysis to provide a reference for the design of the anchoring system.

In order to address the issues mentioned above, this paper investigates the flexural behavior of RC beams strengthened by CFRP with middle anchorage or end anchorage, based on the theory of structural stressing state and number shape function (NSF) method. We modeled the measured strain data as the sum of generalized strain energy density (GSED), to reflect the stress state of the structure. Then the leap characteristic of tested beams’ stressing state was detected by using the Mann–Kendall (M–K) method to the E′–F curves, thereby giving an updated definition of the failure load. In addition, through controlling the value of the pre-tightening torque applied to the anchoring device and the width of the anchoring deformation section, the influence of the pre-tightening torque on the performance of CFRP could also be revealed. Finally, we introduced the NSF method, to expand the limited experimental data and obtain more information about the structural response, to further analyze the force state of the experimental beam.

## 2. The Theory and Methods for Analyzing Structural Stressing State

### 2.1. The Concept of Structural Stressing State to of Beam Model

The concept of structural stress state is essential and gradually accepted by the majority of researchers in structural analysis. However, there is no uniform and precise definition of the complex and continuous structural stress state in the whole loading process so far in the corresponding specifications [24]. Hence, according to the mutual variation and cross-progressive characteristics presented in the structural working process and the structural behavior revealed by classical mechanics, the structural stressing state can be defined as the inner or outer working behavior under a certain loading case; it characterizes the response of different distribution patterns, which reflect the strain, GSED, displacement, deflection, rotation angle, internal force, and other forms of the key points of the structure [25]. In other words, the structural stressing state can be fully represented by numerical model composed of all relevant points mechanical responses.

The main implementation of the structural stressing state theory is to construct a corresponding numerical model to reveal the progressive and qualitative leap characteristics of the mechanical behavior under the entire loading process. Such progressive and qualitative leap features have been verified to conform to the cumulative damage and the natural law from quantitative to qualitative change of a system, and it is a common feature of various structural destruction forms [24]. Moreover, this analysis of structural failure evolution characteristics also establishes a new method for studying the coordination of structural loading period, provides a theoretical basis for engineering to define failure loads and modes, and promotes the rationality of engineering practice.

### 2.2. The Description and Modeling of Structural Stressing State

The structural response of FRP-strengthened RC beams under the action of bending moment was evaluated by using a numerical model. The numerical model collects strain data from experiments as raw data, constructs a GSED numerical model to reflect the stress state of a measuring point, and proposes the parameters of GSED sum, to characterize the stress state of the entire structure [22]. The above method is used to analyze the working behavior of the structure. The specific form is to construct the corresponding characteristic parameters in the form of an array or matrix. Generally, the GSED of a measuring point under a certain load can be calculated by Equations (1) and (2); that is, the generalized strain energy density (GSED), which is taken as a scalar related to strain, can avoid the problem of vector directionality. Here you can directly perform algebraic calculations. For the meaning of specific calculation parameters, please refer to the following:

(1) $$E_{ij}=\int \sigma_{x}d\varepsilon_{x}+\sigma_{y}d\varepsilon_{y}+\sigma_{z}d\varepsilon_{z}$$

where *E_ij_* is the GSED value of the *i*-th measuring point under the *j*-th load; *σ_x_*, *σ_y_*, and *σ_z_* are the nominal stress and *ε_x_*, *ε_y_*, and *ε_z_* are the nominal strain in the three orthogonal directions, respectively.

With reference to the concept of strain energy density, for this paper, we chose generalized (or quasi) strain energy density (GSED) as the characteristic parameter to express the stressing state of the measuring point. Therefore, Equation (1) is simplified to the following:

(2) $$E_{ij}=\frac{1}{2}\sum_{N=1}^{3}E\varepsilon_{N}^{2}$$

where *E_i__j_* is the GSED value of the measuring point, *ε_N_* is the nominal strain in the *N*-th direction, and *E* is the elastic modulus. Moreover, the GSED sum under the *j*-th load obtained by Equation (3) could be used as characteristic parameter to evaluate structural stressing state mode.

(3) $$E_{j}^{\prime }=\sum_{i=1}^{N}E_{ij}$$

where $E_{j}^{\prime }$ is the structural GSED sum under the *j*-th load, and *N* is the total number of measuring points. Furthermore, the $E_{j}^{\prime }-F_{j}$ curve of the structure can be plotted to investigate its stressing state characteristics vividly; here, *F_j_* is the *j*-th load step. The stressing state mode and the corresponding characteristic parameter are a characteristic pair which can reflect the working behavior features of a structure.

### 2.3. The Mann–Kendall Method

According to the natural laws, the damage evolution of the energy system is always a gradual cumulative and progressive process and will eventually change from quantitative to qualitative change. The working behavior of a structure subjected to certain loads must evolve with the increase of load and reflect different stressing state characteristics. In different loading stages, as long as the load reaches a certain level, the stressing state of the structure is likely to show characteristics from quantitative to qualitative change. From the moment of the leap change, the structure has great potential safety hazards and is not suitable for continuing to bearing.

As a hydrometeorological trend test tool, the Mann–Kendall trend test (M–K) criterion of non-distribution test method is used to distinguish the leap and stable characteristics of the structural stressing state. Its advantage is that it does not require samples to obey a certain regular distribution and is not affected by individual outliers [26,27,28]. In this study, the M–K method was utilized to distinguish the mutation load in the ${E^{\prime }}_{j}-F_{j}$ curve. Firstly, define the cumulative number *C_i_* as $C_{i}=\{\begin{array}{cc} +1 & E_{i}>E_{j}(1\leq j\leq i)\\ 0 & otherwise \end{array}$, in which “+1” means adding one more to the existing value if the inequality on the right side is satisfied. Then, a statistical parameter *SP_m_* can be defined as $SP_{m}=\sum_{i=1}^{m}C_{i}(2\leq m\leq n)$, and its mean value and variance can be calculated through $E(SP_{m})=m(m-1)/4$ and $\mathrm{var}(SP_{m})=m(m-1)(2m+5)/72$, respectively. The statistical quantity, *UF_m_*, is defined as $UF_{m}=SP_{m}-E(SP_{m})/\sqrt{\mathrm{var}(SP_{m})}$, to form the *UF_m_* − *F_j_* curve. Subsequently, the same steps are applied to the inverse sequence of $\left\{E^{\prime }\left(i\right)\right\}$, to derive the *UB_m_* − *F_j_* curve. Then the characteristic loads of the $E_{j}^{\prime }-F_{j}$ curve can be determined by the intersection of the *UF_m_* − *F_j_* curve and *UB_m_* − *Fj* curve, as well as the mutation of the structural stressing state.

## 3. Experimental RC Beams

### 3.1. Configuration of Experimental RC Beams

**Figure 1:**
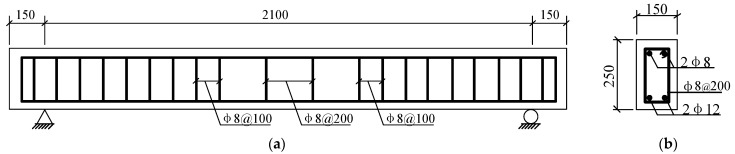
Geometric configuration of the experimental reinforcement concrete (RC) beam and anchorage device (units: mm): (**a**) geometric shape and sizes of the RC beam; (**b**) the reinforcement distribution, shape, and sizes of cross-section.

Chen [29] conducted the experiment of four RC beams strengthened with CFRP by middle anchorage, four RC beams strengthened with CFRP by end anchorage, and one unstrengthened beam. As shown in Figure 1a, these tested RC beams have the same dimensions: 2400 mm in length, 150 mm in width, and 250 mm in height. This test uses commercial concrete with a strength grade of C20; the thickness of the concrete cover is 25 mm. The cement is composed of China Resources brand Portland cement, granite gravel with a particle size of 5–20 mm, tap water, and river sand. The water–cement ratio and sand ratio are 0.41 and 0.33 respectively, and 3% NaCl of cement mass is added during mixing. The concrete mixing ratio is shown in Table 1. The compressive strength value of the cube under standard curing conditions of 28 days is 25.3 Mpa, and the modulus of elasticity is 25.5 GPa. 

The beam’s stirrups and erecting bars are all HPB300-grade smooth round bars with the diameter of 8 mm. In order to prevent the shear failure of the test beam, the stirrups in the shear span area are densified. The stirrup spacing is 100 mm. Since the shear force of the pure bending section is zero due to the constant bending moment, the stirrup spacing is widened to 200 mm; the ribs are HRB400-grade ribbed steel bars with a diameter of 12 mm, and the reinforcement ratio is 0.72%. Figure 1b illustrates the section dimension and the arrangement of steel bars.

The tensile test of CFRP sheet is carried out in accordance with GB/T 3354-1999 “Test Method for Tensile Properties of Oriented Fiber Reinforced Plastics” [30] and STMD3039/D3039M [31], and a tensile test piece of *b_f_* × *t_f_* = 230 mm × 15 mm is made. The steel material property test includes the material properties of the steel bar and anchor steel plate. The above three material property tests of the concrete cube, steel, and FRP sheet are all carried out in accordance with the corresponding specifications. The specific material parameters are shown in Table 2.

### 3.2. Middle Anchorage and End Anchorage System

**Figure 2:**
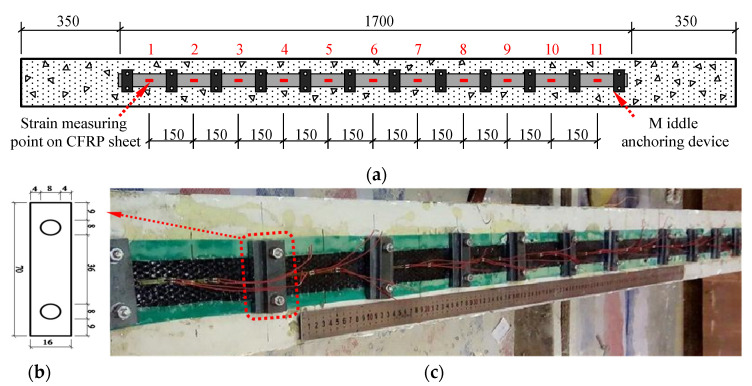
Middle anchoring of CFRP-reinforced specimens (dimensions in mm): (**a**) schematic diagram of middle anchoring and measuring point layout; (**b**) size of middle anchoring device; (**c**) middle anchoring test diagram.

The anchoring system of FRP-strengthened beams in this test includes middle anchorage and end anchorage. In the middle anchoring system, the specific device is shown in Figure 2, and the arrangement of the strain gauge measuring points on the CFRP sheet at the bottom of the beam is shown in Figure 2a; the resistance strain gauge is pasted in the middle of the two adjacent anchors, indicated in red in the figure. As shown in Figure 2c, the expansion bolts are used as the force-generating component. During the loading process, the screw extrusion sleeve produces friction and self-locking to ensure that it is fastened in the concrete body. A 70 mm long, 16 mm wide, and 5 mm thick Q235 steel plate is used as the force transmission device; the specific dimensions are shown in Figure 2b. In this group of experiments, the applied torque was selected as the test variable. By setting three different torques, namely 3.0, 6.0, and 12.0 N·m, the mechanical properties of FRP under different stress conditions were studied, to achieve the adjustment torque, with the purpose of ensuring a higher FRP utilization rate and ductility of the test beam. 

**Figure 3:**
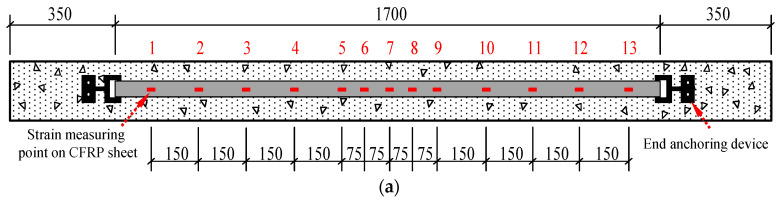

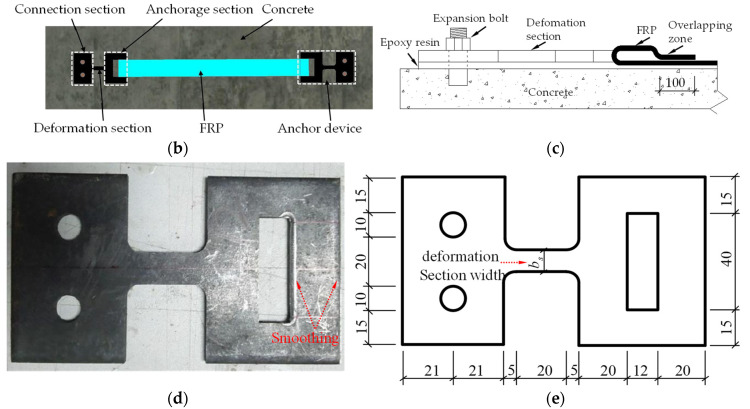
End anchoring of CFRP reinforced specimen (dimensions in mm). (**a**) Schematic diagram of end anchoring and measuring point arrangement. (**b**) End anchoring system. (**c**) Local map of end anchoring connection part. (**d**) Physical drawing of end anchors. (**e**) Dimensions of end anchors.

In the end anchoring system, the arrangement of the CFRP plate strain gauges at the bottom of the beam adopts the method of dense middle and sparse sides. As shown in Figure 3b, within the range of 150 mm on the left and right of the midspan, the distance between the strain gauges is 75 mm, and the distance between adjacent strain gauges in other positions is 150 mm. As shown in Figure 3b, the anchoring device is composed of three parts: anchoring section, deforming section, and connecting section. The specific dimensions and physical pictures of the part are shown in Figure 3d,e. In Figure 3c, the anchoring section mainly anchors the expansion bolts firmly into the concrete body, to ensure that no slippage occurs at the end; the connecting section realizes the connection between the anchor and the FRP. The deformation section is the core part of the technology; by choosing low-grade steel (Q235) and changing the length and cross-sectional area of the deformation section, the FRP working stress level and the FRP–concrete interface slippage can be controlled. In this group of experimental designs, three end anchor deformation section widths of 10, 16, and 20 mm were selected, to study their influence on the control of structural ductility and stress level during FRP peeling.

For RC beams strengthened with CFRP by middle anchorage, three different torques applied on the expansion bolt were taken as test variables. For RC beams strengthened with CFRP by end anchorage, three different widths of the deformation section (***b_s_***) were selected as test variables. Table 3 lists the parameters of RC beams strengthened with CFRP. The system used to number these beams is as follows: “EG” refers to control RC beam strengthened with CFRP, “HG” refers to RC beam strengthened with CFRP by middle anchorage, and “EAG” refers to RC beam strengthened with CFRP by end anchorage.

### 3.3. Loading Scheme and Arrangement of Measuring Points

**Figure 4:**
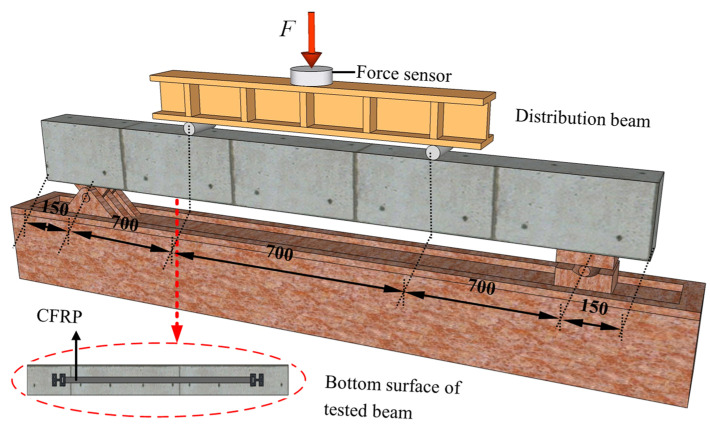
The simplified model of experimental RC beam and loading apparatus.

The schematic diagram of loading devices for the bending test are shown in Figure 4. Two pinned-end supports were arranged at the RC beams’ ends, to simulate the most common end constraints of RC members, resulting in a calculated span of 2100 mm, whereas lengths of pure bending and shear span were both of 700 mm [32,33]. Loads were imposed by using a hydraulic long column tester on the I-beam, to exert loads symmetrically at three dividing points. The load was controlled by applying displacements at a rate of 0.3 mm/min. Six strain gauges were arranged every 50 mm along the cross-section height of the tested beam, to measure the strain of concrete at midspan section. Figure 2a and Figure 3a show the specific layout of the strain gauges for the strain measurement of CFRP bonded on the surface of concrete; data recordings were collected by a Donghua brand DH3823 strain data collection box, and the recording data frequency is 100 Hz (T = 0.01 s). When the crack width of the test beam was too large or shows a significantly larger midspan deformation, the staged loading was stopped, and the beam was directly loaded to failure.

## 4. Stressing State Analysis of RC Beam Strengthened with CFRP by Anchorage Device

### 4.1. Test Results and Load-deflection Curve Analysis

**Figure 5:**
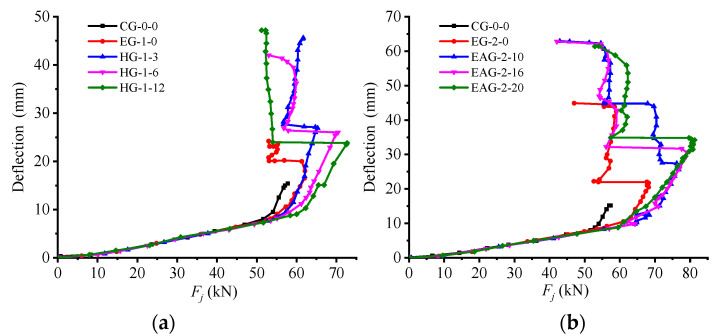
Comparison of load-deflection curves of FRP-reinforced test beams: (**a**) middle anchorage and (**b**) end anchoring.

In order to study the ultimate bearing capacity and ductile deformation capacity of the test beams strengthened by the HG-FRP middle anchor method and the EAG-FRP end anchor method, the midspan load-deflection curve of each test beam during the loading process was drawn and compared with the original The reinforced beam (control group CG-0-0) and the EG-FRP externally bonded beam are compared, as shown in Figure 5a,b. From the comparison of load midspan load-deflection curve, it can be seen that the midspan load-deflection curve of the bending test beam can be divided into three stages before the ultimate load, with cracking point, tensile steel yield point, and peak point as characteristic points The characteristic points of this test correspond to the load, midspan deflection, and ultimate bending moment, and the ductility coefficients are summarized in Table 4. The ductility coefficient is defined as the ratio of the deflection of the lower midspan corresponding to the ultimate load to the midspan deflection of the tensile steel bar when it yields.

It can be seen from Table 4 that the ductility coefficient of the test beams strengthened by the HG-FRP and EAG-FRP methods is significantly greater than that of the unreinforced beam (CG-0-0) and EG-FRP-reinforced beam. It can be seen that the reinforcement by the HG-FRP and EAG-FRP methods is beneficial to improvement The ductility of the unreinforced beam (CG-0-0) and the EG-FRP test beam were analyzed.

By comparing and analyzing the midspan load-deflection curves of different types of test beams in Figure 5a,b, it can be found that, during the entire loading process, before the test beam cracks, the effect of FRP is not reflected. At this time, the concrete in the tension zone plays the major role, the bending crack has not yet occurred, and the shear stress of the FRP concrete interface can be ignored; after the beam cracks, the FRP and the tensile longitudinal reinforcement play a role at the same time before the longitudinal reinforcement yields. Before the longitudinal bars yield and after the beam cracks, the FRP and the tensile longitudinal bars work simultaneously, but the anchors still do not work, and the shear force is mainly transmitted by the binder. After the tensile steel yield point, the load slowly rises, but the midspan deflection increases rapidly. Compared with the unreinforced beam (CG-0-0), the ultimate bearing capacity and ultimate midspan deflection of the FRP-reinforced test beam were greatly improved. This is because the bond between FRP and the concrete matrix makes the stress more evenly distributed and delays the development of cracks, while the ultimate bearing capacity is increased. Moreover, the overall deformation capacity of the beam was also significantly improved.

### 4.2. Characteristic Parameter of HG-1-12 RC Beam’s Stressing State

**Figure 6:**
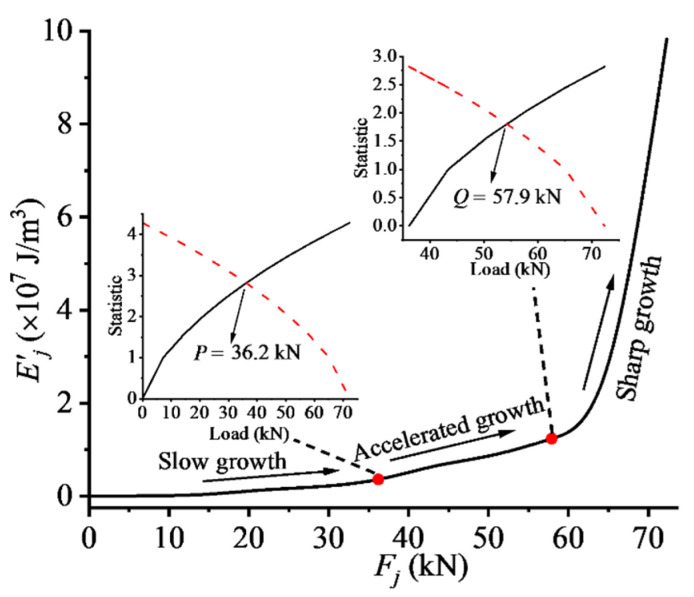
The HG-1-12 RC Beam’s $E_{j}^{\prime }\;-\;F_{j}$ curve and M–K statistic curves: P = 36.2 kN and Q = 57.9 kN are the characteristic loads detected by M–K criterion.

In order to characterize the changing process of structural stressing state synthetically, the GSED sum taking all the measuring strain data of key points into consideration is established as a characteristic parameter. For the RC beam numbered HG-1-12 exampled here, the GSED sum ($E_{j}^{\prime }$) at each load step can be calculated through Equation (3). Then the $E_{j}^{\prime }-F_{j}$ curve can be plotted, to investigate the working behavior of the RC beam. Two characteristic loads, P = 36.2 kN and Q = 57.9 kN, determined by applying M–K criterion to the $E_{j}^{\prime }-F_{j}$ curve, divide the whole working performance into three stages, as shown in Figure 6. (1) Between the load 0–P, the sum of the GSED value, $E_{j}^{\prime }$, slowly increases with load, showing a stable change. Micro-cracks in the beam model and the gap between the aggregates in the initial loading stage indicate a steady change of the strain value of measuring points. The RC beam could basically maintain a stable stressing state due to the fact that the CFRP and steel bars are in linear-elastic working state. (2) From load P to Q, local yield of steel bars and development of concrete cracks, the structure reaches the elastic–plastic stress state; however, the value of the GSED sum continues to increase in a relatively stable manner, and its growth accelerates to some extent with the development of concrete cracks. Meanwhile, the CFRP sheet exerts its effect to strengthen the specimen, and the RC beam enters elastic–plastic working state until Q. (3) After the load Q, the growth trend of $E_{j}^{\prime }$ changes sharply with concrete cracking and the yield of steel bars, displaying that the stressing state of the RC beam changes from being in a stable state to an unstable state, until the ultimate load (U = 72.3 kN) finally fails.

Therefore, the characteristic load Q reveals the starting point of structural failure process and is defined as the failure load of the structure. It updates the exist definition of failure load and has different meaning with the present failure load, which is uniquely called the ultimate load. The updated failure load Q would reflect the normal service of the structure factually and the leap characteristic of structural stressing state, in accordance with the natural law from quantitative change to qualitative change of a system. In addition, it would also provide a more accurate and rational design reference. Moreover, the stressing state mode needs to be further be investigated to verify the stressing state characteristics embodied in the $E_{j}^{\prime }-F_{j}$ curve.

### 4.3. Investigation of Tested RC Beam’s Stressing State Mode

**Figure 7:**
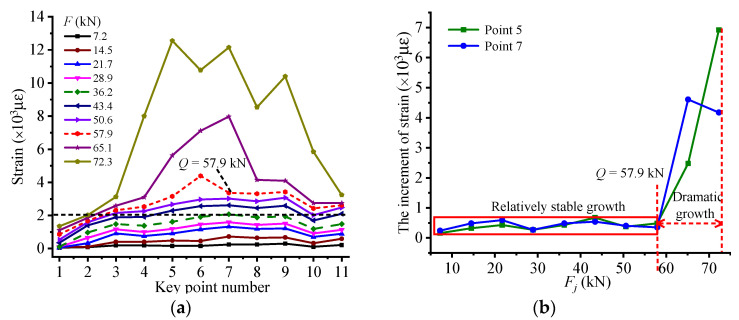
The changing features of strain-based stressing state mode: (**a**) the strain distribution pattern of CFRP bonded on the bottom surface of HG-1-12; (**b**) the increment of strain at measuring points 5 and 7.

The strain at the measuring point represents the stressing state of this point. Therefore, strain values of all measuring points on the structure could be modeled to evaluate the structural stressing state. For the HG-1-12 beam, the strain data of CFRP bonded on the bottom surface of specimen are recorded to investigate the working performance of RC beam and reinforcement effect of CFRP with middle anchorage. As shown in Figure 7a, the strain distribution pattern of CFRP is plotted, and it can be seen that the strain of CFRP on both sides of the midspan axis is basically symmetrical. The large strain value of CFRP in the range of −450 to 450 indicates that this part of CFRP is fully utilized. In addition, before 36.2 kN, the strain of measuring point on the CFRP sheet is less than 2000 με, and the distribution pattern is relatively uniform, showing the outstanding coordination working behavior of the RC beam and excellent load-transmitting performance between concrete and CFRP. With the load continuing to increase, the strain values of the CFRP near midspan rise faster those that at the ends of the CFRP. Especially, when the load exceeds 57.9 kN (load Q), the strain of measuring points turns out to dramatic growth and transfers to two sides fast, compared with previous stage with relatively stable growth, indicating that a leap characteristic is embodied in the structural stressing state at failure load. Figure 7b demonstrates the strain increment at measuring points 5 and 7, which corresponds pretty well with the analysis conclusion. What is more, the maximum strain value of CFRP reaches 12,555 με, close to its facture strain at ultimate load, showing that the anchor device could fully develop the effect of CFRP and greatly improve its utilization rate. It should be highlighted that the regions where CFRP plays a significant role are mainly located from point 4 to point 8.

### 4.4. Stressing State Mode Analysis of RC Beams with Middle Anchorage

**Figure 8:**
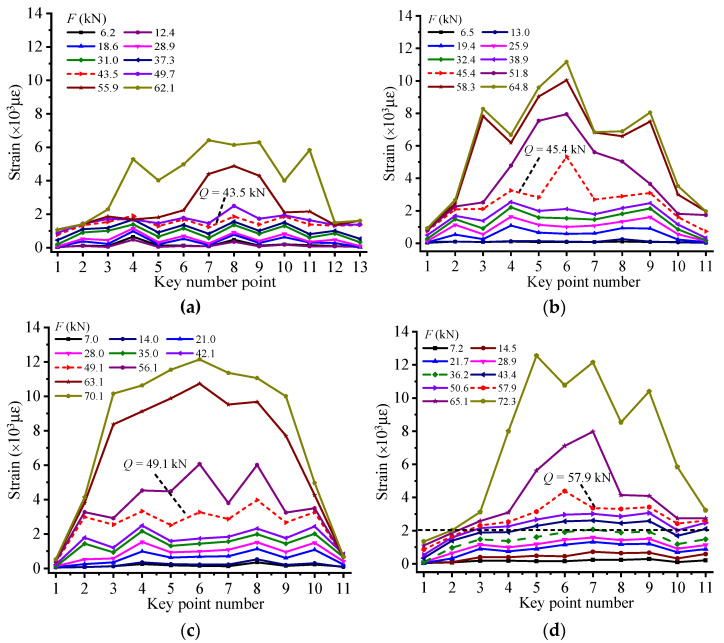
Strain distribution pattern of CFRP for (**a**) EG-1-0, (**b**) HG-1-3, (**c**) HG-1-6, and (**d**) HG-1-12.

As illustrated in Figure 8, the strain distribution pattern of CFRP is plotted to analyze the effect of torsion applied on the anchorage device on the RC beam’s working performance. Moreover, the dash lines marked in the figures are respective failure loads of experimental beams determined by applying the M–K criterion to their $E_{j}^{\prime }\;-\;F_{j}$ curves. Obviously, the leap characteristics under failure loads are vividly embodied in these strain distribution patterns, showing that a substantial change occurs in RC beams’ stressing state and verifying the accuracy of failure loads detected by M–K criterion. Through comparing the strain of CFRP of the RC beams with middle anchorage or without anchorage device, some research results can be summarized as follows:

- For the RC beam numbered EG-1-0, after failure load, the bond force between the concrete and CFRP could not be effectively transferred to the ends of RC beam because of the peeling between these two materials. The strain of CFRP near the midspan section increases significantly, while the strain at the ends of CFRP sheet rises slowly. When the experimental RC beam is destroyed, the maximum strain value of the CFRP only reaches 6289 με, showing that the reinforcement effect of CFRP is not fully exerted and the composite action between CFRP and concrete is not fully exerted.
- Compared with the control beam (EG-1-0), the failure load of the RC beam with middle anchoring was improved to a certain extent. With the increase of the torsion applied to the anchoring device, the failure load of the RC beam also gradually increases, which indicates that the arrangement of middle anchorage on CFRP could effectively delay the peeling of the CFRP–concrete interface, thereby providing a firm mutual interaction between CFRP and concrete. The application of an anchoring device can give full play to the role of CFRP and further improve the bending performance of the tested reinforced concrete beam.
- Since the ability of the adhesive to transfer stress depends on its bond with the concrete and CFRP and the interfacial shear stresses, the scheme of setting middle anchorage on the CFRP could increase the positive stress and improve the bond force of interface, so that the CFRP sheet can be efficiently used near the ends. As shown in Figure 8, the maximum strain of CFRP of RC beams with middle anchorage is almost twice that of EG-1-0. In addition, as shown in Figure 8c, compared with other experimental RC beams, the CFRP strain distribution diagram of HG-1-6 represents the maximum utilization range of CFRP with high strain levels from point 2 to point 10.

### 4.5. Stressing State Mode Analysis of RC Beams with End Anchorage

**Figure 9:**
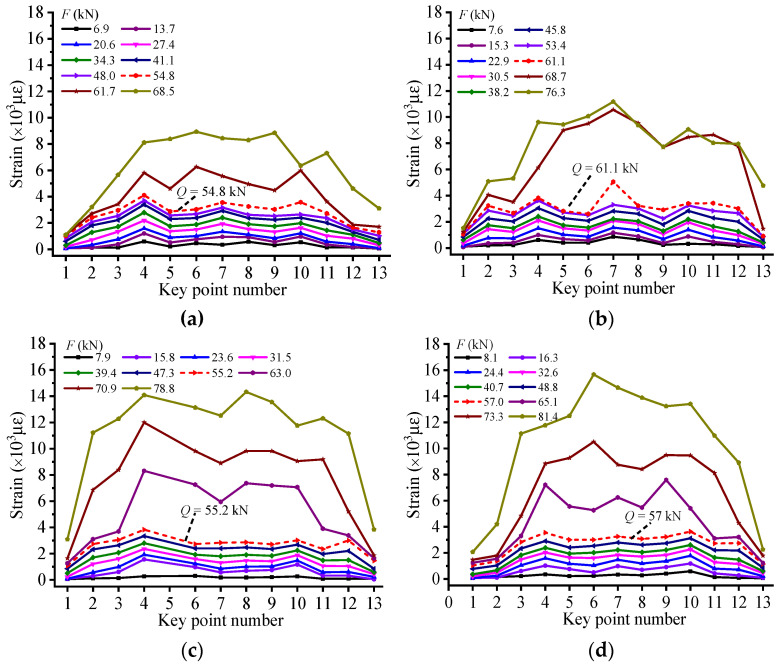
The strain distribution pattern of CFRP for (**a**) EG-2-0, (**b**) EG-2-10, (**c**) EG-2-16, and (**d**) EG-2-20.

For experimental RC beams with end anchorage, the width of the deformation section is set to 10, 16, and 20 mm, respectively, to evaluate the influence of the anchoring device on the CFRP reinforcement effect. Figure 9a shows the CFRP strain distribution of a controlled RC beam without end anchors. Figure 9b–d respectively shows the CFRP strain distribution patterns of RC beams applied by end anchors with different deformation section widths. respectively. Meanwhile, the respective failure loads of specimens distinguished by applying M–K criterion to their $E_{j}^{\prime }\;-\;F_{j}$ curves are marked by short red dashed lines. It can be seen from Figure 9 that the strain of CFRP at each measuring point of the tested beams rises steadily before their failure loads. Moreover, the strain value of each measuring point at the CFRP sheet shows a dramatic increase after failure loads, verifying that an apparent leap characteristic occurs in the beam’s stressing state. 

In addition, for the beam numbered EG-2-0, before its failure load, the strain distribution of CFRP sheet is uniform, which shows that the shear force can be transferred well at the interface between concrete and CFRP. However, the CFRP starts to peel from the bottom of concrete after failure load, due to the lack of effective anchorage. Moreover, the interface debonding of CFRP takes place near the midspan section firstly and extends to the end of CFRP sheet quickly, resulting in complete debonding of CFRP from the bottom of beam and rapid loss of RC beam’s bearing capacity. The maximum strain value of CFRP under ultimate load is only 8900 με, indicating that the strengthening performance of CFRP is developed with lower utilization. 

For the beam numbered EG-2-10, the application of end anchorage could efficiently avoid the peeling of CFRP, to further promote the strengthening performance of CFRP. Moreover, the failure load of EG-2-10 and ultimate strain value of CFRP increase greatly compared with those of EG-2-0. Nevertheless, the deformation section of the end anchorage device is only 10 mm in width, which is too narrow to provide sufficient strength in order to restrict the peeling of CFRP. The RC beam finally loses its bearing capacity due to the fracture of deformation section. 

Appropriately increasing the width of the deformed portion can be beneficial to improve the reinforcement performance of CFRP. By studying the CFRP strain distribution of EG-2-16 and EG-2-20, the maximum strain of CFRP reached 14,327 and 15,666 με, respectively, which was close to its breaking strain. This shows that CFRP has played almost all functions in strengthening RC beams. Since the deformed section of the end anchoring device has sufficient width to provide effective restraint, the two RC beams lose their bearing capacity due to the fracture of the CFRP in the midspan section. The sample numbered EG-2-16 has the largest CFRP high strain level range from point 4 to point 12 than the rest of the RC beam, indicating that the CFRP bonded to the bottom of the RC beam has been fully utilized.

## 5. The Application of Numerical Shape Function in the Stressing State Analysis of Experimental RC Beam

### 5.1. The Method of Numerical Shape Function

**Figure 10:**
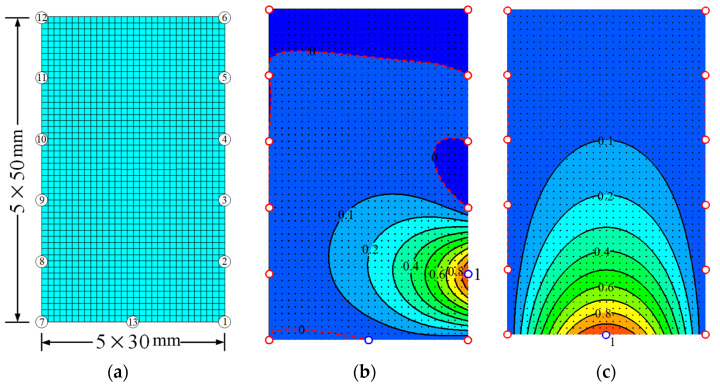
Finite element model and contour map of numerical shape function. (**a**) Finite element model. (**b**) Shape function ***N_2_***. (**c**) Shape function ***N_13_***.

Due to the limitation of existing measuring instruments or high costs, the measured experimental data are not sufficient to fully present the structural response mechanism and working performance. Although many of space interpolation methods have been proposed to predict the unsampled points’ data, the estimation accuracy seems difficult to meet the requirements due to ignoring the invisible information between spatial distribution and numerical values of samples. Moreover, the usage of space interpolation methods is constrained to some extent because of excessive assumptions on the mathematical models and corresponding parameters [34]. Therefore, the numerical shape function (NSF) interpolation method with clear physical meaning is proposed to expand the limited experimental data and make up for the defects of tradition interpolation methods. The construction process of NSF is given as follows:

- As shown in Figure 10a, for the finite element model of RC beam’s midspan section was created with ANSYS 14.0 software. Shell 181 element is adopted for concrete with 5 mm in thickness and has an area of 5 × 5 mm^2^. Beam 188 element is used for CFRP in the simulation of cross-section. Its thickness is also 5 mm, and its length takes the real width of the CFRP sheet. Beam 188 element is also applied for longitudinal reinforcement. Its area takes the actual area of longitudinal reinforcement, and its thickness is 5 mm. In addition, the connection between reinforcement and concrete is assumed to be rigid. Strain values of 13 key points in the cross-section are measured as samples, to predict strain field of midspan section.
- Each shape function, **N*_i_***, of the *i*-th point (*i* = 1, 2, 3, …, 13) should be prepared in advance, to obtain the final interpolated field of each load step. For instance, as shown in Figure 10b, the shape function **N_2_** of point 2 could be derived from finite element simulation by exerting displacement *d* = 1 at point 2, marked by a blue circle, and *d* = 0 at the other key points, marked by a red circle. Moreover, the form of **N_2_** is $N_{2}=[N_{2}(d_{1}),N_{2}(d_{2})\cdots N_{2}(d_{j})\cdots N_{2}(d_{n})]$, where *N_2_*(*d_j_*) is the simulated value at element node *d_j_*, and *n* is the total node of the cross-section. Moreover, Figure 10c shows the shape function of **N_13_** obtained by the same steps.
- The interpolated field of cross-section model can be calculated by the linear combination of shape functions obtained above, as follows:
  (4) $$D=\sum_{i=1}^{m}u_{i}N_{i}$$
  where D is the interpolated field of cross-section model; *u_i_* is the measuring samples; m is the total number of measuring points, and it is 13 in this case.

### 5.2. Accuracy Verification of Expanded Experimental Data

In order to verify the accuracy of the proposed NSF method, 11 measuring points are applied to establish the strain field of midspan section. Then interpolated strain value of the left key point could be obtained from checking the node data of the same location in the strain field. The interpolating data of the key point and its actual measuring data could be placed in the same graph, to evaluate the accuracy of NSF method. In addition, the error, *δ_i_*, under the *i*-th load and the average error, $\bar{\delta }$, of key point can be calculated by Equations (5) and (6):

(5) $$\delta_{i}=\left|\frac{\varepsilon_{i}-\varepsilon_{e}}{\varepsilon_{e}}\times 100\%\right|$$

(6) $$\bar{\delta }=\frac{1}{N}\sum_{i}^{N}\delta_{i}$$

where *ε_i_* and *ε_e_* are the interpolating and experimental strains, respectively; and *N* is total number of load steps.

The second aspect is cross-validation. The validation errors of 13 measurement points in 10 load stages are listed. Here, the leave-one-out (LOO) cross-validation method [35] is used; that is, in the *j*-th load step, all the strain data except the *i*-th measuring point are used. The NSF method is applied to construct the cross-sectional strain field; for the *i*-th measurement point, compare the error between the interpolation data and the test data.

**Figure 11:**
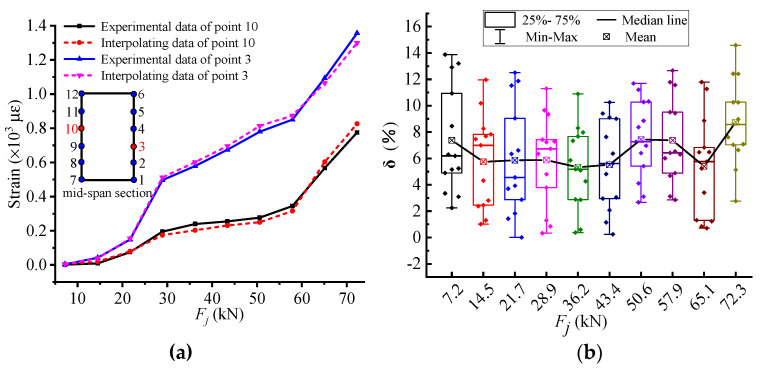
Strain data error results of the HG-1-12 specimen midspan section. (**a**) Comparison curve for points 3 and 10 on the midspan section. (**b**) Error of cross-checking of all measuring points.

As illustrated in Figure 11a, the comparisons of the interpolating strains with the experimental ones at the comparative position are marked by red circle. The interpolating data almost coincide with the experimental data in the whole loading process. Moreover, the maximum and average errors of point 3 are 7.31% and 4.1%, respectively. Hence, it is available to apply the NSF method to expand the experimental data.

Figure 11b shows the strain error of all measuring points verified by LOO. The overall error is in the range of 0–15%, and the average error is 7.71%. The error result is relatively small and fully meets the application requirements. Meanwhile, the error is relatively large in the initial stage of loading and after the "failure" load Q. This is because the slight difference at the initial loading stage is amplified by the smaller denominator in the error Equation (5), and the "failure" load Q after the strain mutation characteristics lead to an increase in the error of NSF calculation. Through the above analysis and comparison, the NSF method can be used to expand the experimental data with sufficient accuracy to deeply explore the potential characteristics of the stressing state of the RC beam strengthened by CFRP sheet.

### 5.3. Comparison between Curves Obtained by Two Methods

The midspan section of the tested RC beam was meshed by the finite element method. By applying the NSF method, the strain of all nodes can be interpolated, and corresponding stress values are calculated through the constitutive relation of the material. Hence, the GSED values of cross-section can be expressed by the following equations:

(7) $$e_{ij}=\int_{0}^{\varepsilon_{j}}\sigma_{i}d\varepsilon_{i},\;E_{j}=\sum_{i=1}^{N}e_{ij}A_{i}$$

where *e_ij_* is the GSED value of the *i*-th element at the *j*-th load step; *σ_i_* is the normal stress of the *i*-th element; *ε_i_* is the normal strain of *i*-th element; *ε_ij_* is the normal strain of the *i*-th element at the *j*-th load step; *E_j_* is the GSED value of the midspan section at the *j*-th load step; *N* is the total number of elements; *A_i_* is the area of the *i*-th element.

**Figure 12:**
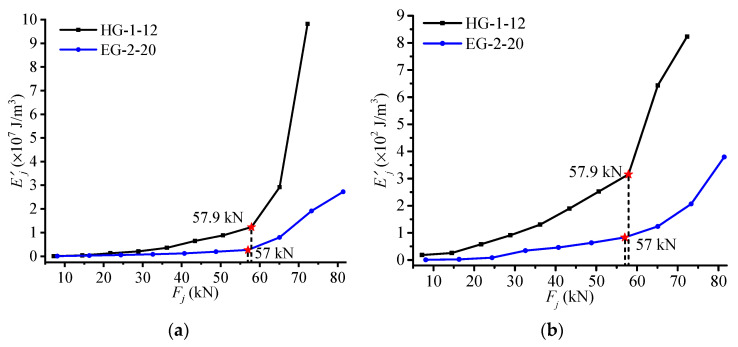
(**a**) The $E_{j}^{\prime }\;-\;F_{j}$ curves using tested strains. (**b**) The $E_{j}^{\prime }\;-\;F_{j}$ curves using expanded strains.

Therefore, the $E_{j}^{\prime }-F_{j}$ specimen curves of HG-1-12 and EG-2-20 can be plotted by applying the proposed NSF method, as shown in Figure 12b. The experimental strain data of these two specimens can also be directly used for calculating their GSED values of midspan section based on Equations (2) and (7) for comparison. Then corresponding $E_{j}^{\prime }-F_{j}$ curves can be derived as shown in Figure 12a. It can be seen that two groups of curves in Figure 12a,b embody the similar tendency and changing features: (1) After RC beams’ respective failure load detected by the M–K criterion, the GSED values show dramatic growth, as compared with the previous stage. (2) The failure load of HG-1-12 is larger than that of EG-2-20, indicating that the middle anchored CFRP sheet has a better reinforcement effect than the end anchored CFRP sheet. Accordingly, all of these proofs illustrate that the structural stress state analysis based on the NSF method can reveal the working features of RC beams.

### 5.4. Analysis of Strain Fields’ Changing Characteristics

**Figure 13:**
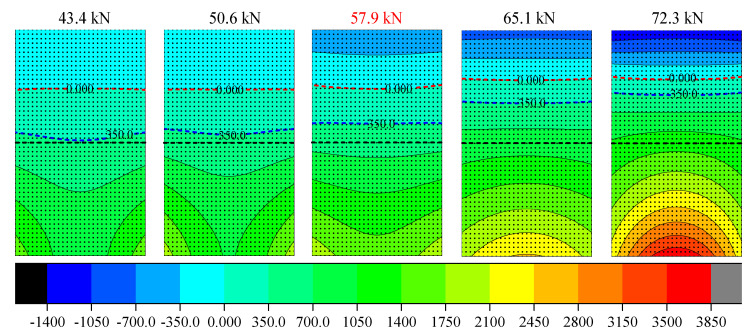
The strain contour maps of HG-1-12.

For the test specimen HG-1-12, the midspan section bears the maximum bending moment and serves as the design control section; its structural stressing state features may embody the response of midspan section to some extent. Hence, the midspan section is determined as a key section to investigate the changing characteristics of strain fields, which can be obtained by interpolating the measured strain by using the NSF method. As shown in Figure 13, five strain contour maps of HG-1-12 around its failure load are plotted with the same color scale in order to vividly present the changing process of strain distribution. The contour line in the figure represents the regional distribution of concrete strain, which is convenient for observing its changing law. In addition, in order to intuitively distinguish the tensile and compressive region of cross-section, a red short dashed line is used to mark the 0με boundary. The dividing line of 350 με (approximate ultimate tensile strain) with blue short dash line is used to evaluate the range of areas beyond ultimate tensile strain. It can be seen from Figure 13 that the boundary of tensile strain gradually rises with load increasing, indicating the area of tensile zone expands with the development of concrete cracks. Meanwhile, before 57.9 kN, the separatrix of 350 με rises much slower and is far from the dividing line of 0 με. When the load exceeds 57.9 kN, the region where the concrete strain is beyond 350 με dramatically increases and the boundary of 350 με gradually approaches the separatrix between tensile strain and compressive strain of the cross-section. Furthermore, through comparing these five contour maps around the failure load, a significant increase in the number of levels of contour lines can be observed intuitively with the increase of loads, especially after the failure load. It also can be seen from Figure 13 that the contour lines below the black dash line are concave before 57.9 kN with low strain level in the central of section and high strain level at the edge of section, due to the effective reinforcement of CFRP sheet with valid middle anchorage. However, after 57.9 kN, the contour lines below the black dash line show opposite distribution characteristics with the previous contour maps, which demonstrates that the CFRP sheet cannot provide effective strengthening for the RC beam, due to its debonding from the concrete surface. On all of these counts, there is a large difference in the strain distribution pattern of contour maps before and after 57.9 kN, indicating that the failure load defined is the qualitative mutation load of structural stressing state. Besides this, it also verifies that the RC beam’s stressing state features are embodied in the changing trend of its strain fields, according to the above analysis.

## 6. Conclusions

To evaluate the effect of the anchoring device on the strengthening performance of CFRP sheets, an investigation on RC beams strengthened by CFRP with different anchorage schemes was conducted. The structural stressing state theory was used to reveal the changing characteristics of experimental RC beams’ working behavior under bending moment. Applying the M–K criterion, the qualitative leap features of tested beams’ stressing state can be detected, which can be seen as the starting point of RC beam’s failure and leads to the update of the existing definition of failure load. The achieved results can be concluded as follows:

- For experimental specimens with middle anchorage, the strain distribution of CFRP was analyzed to evaluate the effect of torsion applied on the anchoring device on the reinforcement performance of the CFRP sheet. Combined with respective failure loads, its effect on the working behavior of RC beams can be further illustrated. In this study, when the torsion is set to 6 kN·m, the strain distribution of CFRP is more uniform, and its strengthening performance can be fully developed.
- For experimental specimens with end anchorage, through investigating the strain distribution of CFRP, the effect of deformation width of anchoring device on the reinforcement performance of CFRP sheet can be revealed. Then its effect on the RC beams’ working features can also be stated according to their failure loads. In this study, when the deformation width was set to 16 mm, the strengthening performance of CFRP was fully developed and greatly improved the ability of RC beams in resisting the bending moment.
- With the application of the NSF method, the limited strain data were reasonably expanded to further explore the changing features of strain field, which vividly revealed the changing characteristics of the RC beam’s stressing state. It also provided a new angle of view to predict sectional response information and conduct structural analysis.

## Figures and Tables

**Table 1:** C20 concrete mix ratio.

Strength Grade	Water (kg)	Cement (kg)	Sand (kg)	Crushed Stone (kg)
C20	180	439	587.7	1193.3

**Table 2:** The properties of steel bar, steel plate, and Carbon-Fiber-Reinforced Plastic (CFRP).

Material	Yield Stress (MPa)	Fracture Strain (με)	Fracture Strength (MPa)	Elastic Modulus (GPa)
Steel bar	454.11	\	620.45	212.13
Steel plate	291.28	\	423.26	204.9
CFRP	\	16,753	4103.4	270.45

**Table 3:** Specimen numbers and strengthening configurations.

Middle Anchorage					End Anchorage			
Specimen Number	CFRP Layer Number	Torque (kN·m)	Space (mm)	Number	Specimen Number	CFRP Layer Number	Steel Plate Thickness (mm)	Deformation Width-*b_s_* (mm)
CG-0-0	0	/	/	/	EG-2-0	2	0	0
EG-1-0	1	0	0	0	EAG-2-10	2	5	10
HG-1-3	1	3	150	12	EAG-2-16	2	5	16
HG-1-6	1	6	150	12	EAG-2-20	2	5	20
HG-1-12	1	9	150	12				

**Table 4:** Specimen numbers and strengthening configurations.

Specimen Number	Cracking Point		Yield Point		Peak Point			Ductility Coefficient
	Midspan Deflection (mm)	Cracking Load (kN)	Midspan Deflection(mm)	Yield Load (kN)	Midspan Deflection (mm)	Ultimate Load (kN)	Limit Moment (kN.m)	
CG-0-0	2.01	13.80	7.69	50.84	15.31	57.6	20.21	1.99
EG-1-0	1.58	14.95	8.65	51.58	19.85	62.1	21.77	2.29
HG-1-3	1.47	15.18	8.46	54.88	26.99	64.8	22.75	3.19
HG-1-6	1.37	15.77	8.14	55.26	26.02	70.1	24.6	3.2
HG-1-12	1.28	15.28	8.92	58.63	23.76	72.3	25.37	2.66
EG-2-0	1.34	14.26	10.03	60.81	22.06	68.5	24.04	2.2
EAG-2-10	1.4	14.84	10.05	64.21	27.09	76.3	26.76	2.7
EAG-2-16	1.32	14.37	9.99	60.95	34.98	78.8	27.62	3.5
EAG-2-20	1.39	14.49	9.93	61.47	34.29	81.4	28.55	3.45

## Data Availability

Data is contained within the article.
